# Exogenous epibrassinolide application improves essential oil biosynthesis and trichome development in peppermint via modulating growth and physicochemical processes

**DOI:** 10.1038/s41598-023-40210-9

**Published:** 2023-08-09

**Authors:** Zubair Ahmad Parrey, Sajad Hussain Shah, Firoz Mohammad, Manzer H. Siddiqui, Saud Alamri, Hazem M. Kalaji

**Affiliations:** 1 Plant Physiology and Biochemistry Section, Department of Botany, Aligarh Muslim University, Aligarh, 202002 India; 2 Department of Botany and Microbiology, College of Science, King Saud University, Riyadh, 11451 Saudi Arabia; 3 Department of Plant Physiology, Institute of Biology, Warsaw, University of Life Sciences SGGW, Nowoursynowska 159, 02-776 Warsaw, Poland

**Keywords:** Plant sciences, Plant physiology

## Abstract

Peppermint has gained a promising status due to the presence of a high proportion of bioactive compounds, especially menthol. Due to its pharmacological efficacy, the demand for its plant-based bioactive compounds necessitates its cultivation worldwide. Brassinosteroids are polyhydroxylated sterol derivatives that regulate diverse processes and control many agronomic traits during plant growth and development. A factorial randomised pot experiment was performed in the net house to investigate the effect of 24-Epibrassinolide (EBL) on the growth, physiology, essential oil content, stomatal behaviour and trichome development of the three cultivars of peppermint. Four levels of foliage-applied EBL, viz. 0, 10^–5^, 10^–6^ and 10^–7^ M were applied to the three cultivars of peppermint (Kukrail, Pranjal and Tushar). Among the different treatments of EBL, the application of 10^–6^ M increased shoot length by 38.84, 37.59 and 36.91%, root length by 36.73, 29.44 and 33.47%, chlorophyll content by 24.20, 22.48 and 23.32%, P_*N*_ by 32.88, 32.61 and 33.61%, EO content by 32.72, 30.00 and 28.84%, EO yield per plant by 66.66, 77.77 and 73.33% and menthol yield per plant by 127.27, 110 and 118.18% in Kukrail, Tushar and Pranjal respectively, compared with their respective control plants. Further, the 10^–6^ M EBL exhibited improved trichome size and density, cellular viability and menthol content of the oil analysed from scanning electron microscopy, confocal laser scanning microscopy and GC–MS respectively as compared to the control. In conclusion, out of different levels of EBL, two sprays of 10^–6^ M EBL proved effective in enhancing the morphophysiological features and productivity of mint plants, particularly for cultivar Kukrail.

## Introduction

*Mentha piperita* L. (peppermint) is an important medicinal and aromatic herb grown all over the world. Its leaves are used in herbal tea and as a spice while its essential oil (EO) is widely employed in pharmaceutical, cosmetic, food, cleaning and personal care for both flavouring and fragrance properties^1^. Moreover, peppermint oil has several biological properties including antifungal, antimicrobial, cytotoxic and antibacterial that markedly increase its high economic value in the world market for the EO industries^2, 3^. Peppermint oil is abundant in secondary metabolites, among which menthol stands out as the principal constituent. Additionally, notable compounds present in peppermint oil include menthone, menthofuran, and pulegone. These diverse secondary metabolites collectively contribute to the aromatic profile and therapeutic attributes associated with peppermint oil. On the other hand, its EO contains menthofuran and pulegone which are undesirable components due to their hepatotoxic nature. So, it is very important to have a high proportion of menthol and low content of menthofuran and pulegone which improves the quality of the peppermint oil^4^. In the present era, the cultivation, production and supply of EOs lag behind their demand in the market and are believed to increase further in the near future. As market demand continues to rise, there is an imperative to enhance peppermint EO production to meet the needs of various industries, especially in the wake of the global impact of the Coronavirus Disease 2019 (COVID-19) pandemic. Aromatherapy and personal care sectors worldwide have experienced a significant hike in demand for EOs, making it essential to address this rising market pull. Consequently, large-scale modulation and enhancement of EO production in peppermint become crucial in bridging the gap between supply and demand and it expectedly address the ever-growing market requirements^5, 6^. In this regard, several methods have been adopted from time to time ranging from the exogenous application of mineral nutrients to plant growth regulators to explicate their potential in increasing the EO content and modulation of EO components in medicinal and aromatic plants^7–11^.

Brassinosteroids are polyhydroxylated sterol derivatives ubiquitously present in all plants^12^. They regulate diverse processes and control many agronomic traits during overall plant growth and development. They play a key role in plant photomorphogenesis, seed germination, root proliferation and development, stomatal development, cell elongation and cell division, vascular differentiation, photosynthesis, enzyme activation, gene regulation, protein and nucleic acid synthesis, flowering, seed yield and fruit set^13–15^. Apart from these promoting effects, the biosynthesis of specialized metabolites such as EO biosynthesis and its compositional modulation of the oil in aromatic and medicinal plants are known to be modulated by the application of brassinosteroids as they are also known to regulate the biosynthesis of secondary metabolites viz., EOs in plants^8^.

In light of the growing demand for peppermint EO and the significance of brassinosteroids in modulating the plant morphophysiological process and biosynthesis of specialized metabolites (essential oil) that collectively would enhance the quantity and quality of medicinal and aromatic plants. The present study was designed to examine the effect of foliar application of different levels of 24-epibrassinolide (EBL) on growth, physio-biochemical attributes, EO biosynthesis and stomatal and trichome behaviour of the selected cultivars of peppermint.

## Results

The treatment effects and their interactions with cultivars were found to be significant on all examined parameters, with the exception of leaf potassium (K) and phosphorus (P) content, nitrate reductase (NR) activity, stomatal conductance (*gs*), stomatal aperture length and width, and trichome density, for which the interaction effect was not significant. Among the foliar spray treatments, the T3 treatment yielded the highest value, followed by the T4 treatment. However, the cultivar Kukrail performed best and was followed by the cultivar Tushar.

### Epibrassinolide modulates growth-related traits of peppermint

**Figure 1:**
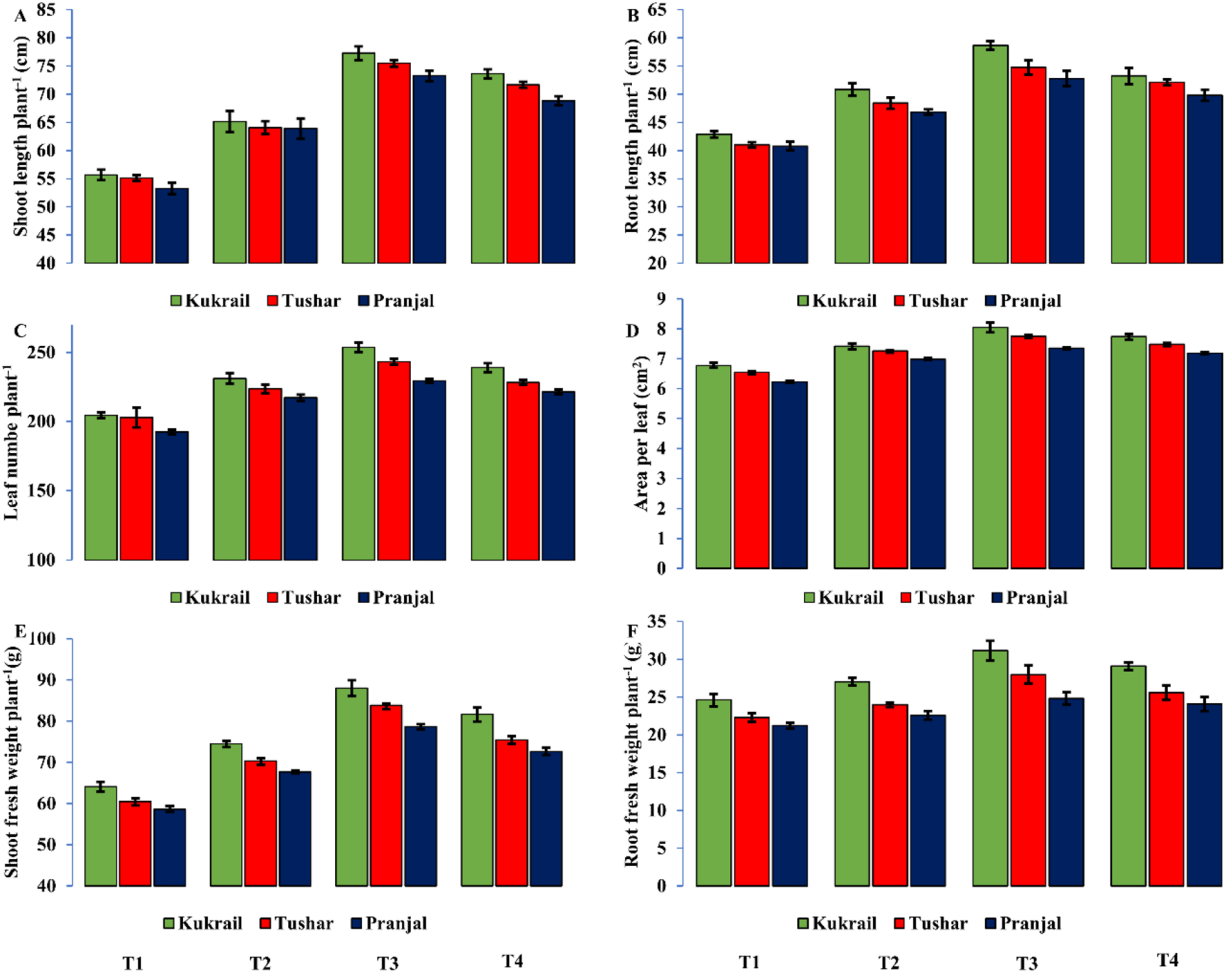
Effect of different concentrations of EBL on: (**A**) Shoot length per plant (**B**) Root length per plant (**C**) Leaf number per plant (**D**) Area per leaf (**E**) Shoot fresh weight per plant (**F**) Root fresh weight per plant of peppermint cultivars Kukrail, Pranjal and Tushar. Columns represent the mean and bars represent the standard error (SE) of four replicates of each cultivar.

The growth-related traits were enhanced with the application of EBL in all three cultivars studied. The application of 10^–6^ M EBL enhanced the shoot length per plant by 38.84, 37.59 and 36.91%, root length per plant by 36.73, 29.44 and 33.47%, leaf number per plant by 24.08, 19.22 and 19.97%, area per leaf by 18.90, 18.16 and 18.50%, shoot fresh weight per plant by 37.27, 34.20 and 38.74% and root fresh weight per plant by 26.52, 16.96 and 25.42% in Kukrail, Tushar and Pranjal respectively over their respective water spray treatments (Fig. 1A–F).

### Epibrassinolide regulates the physiobiochemical attributes and mineral elements of peppermint

**Figure 2:**
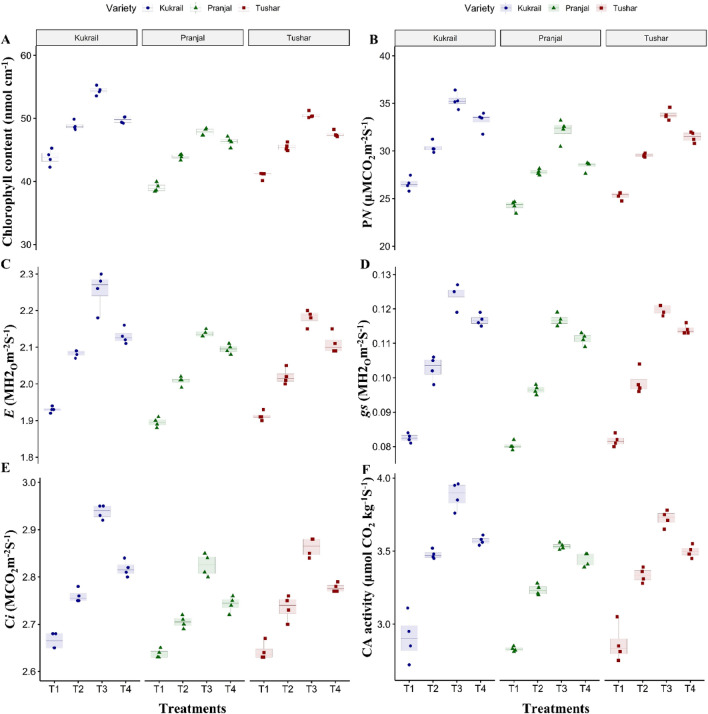
Effect of different concentrations of EBL on: (**A**) Chlorophyll content (SPAD) (**B**) Net photosynthetic rate (**C**) Transpiration rate (**D**) Stomatal conductance (**E**) Intercellular carbon dioxide concentration (**F**) Carbonic anhydrase activity of peppermint cultivars Kukrail, Pranjal and Tushar. The different shapes in the box plot represent the mean of four replicates of each cultivar.

**Figure 3:**
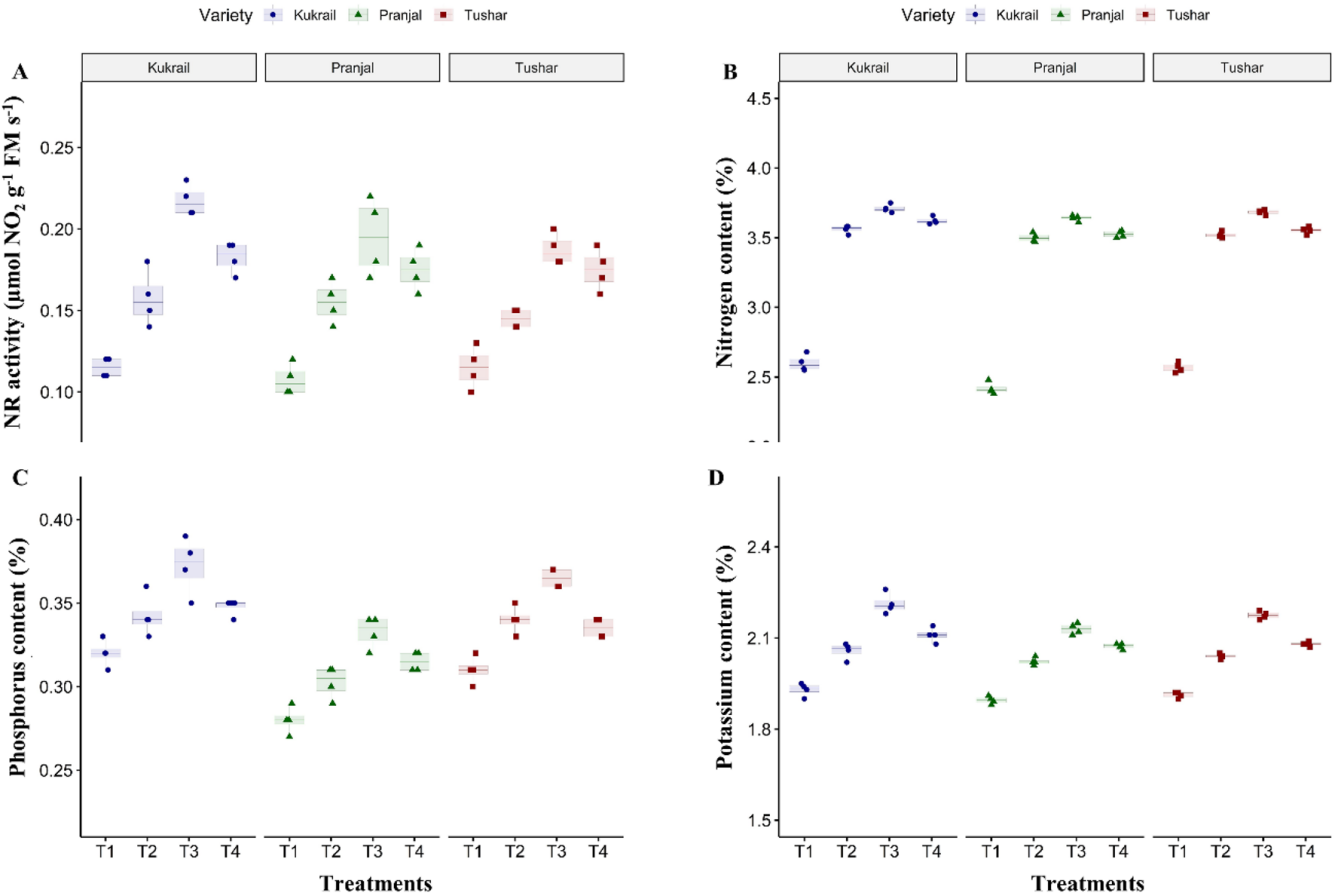
Effect of different concentrations of EBL on: (**A**) NR activity (**B**) Nitrogen content (**C**) Phosphorus content (**D**) Potassium content of peppermint cultivars Kukrail, Pranjal and Tushar. The different shapes in the box plot represent the mean of four replicates of each cultivar.

The foliar application of 10^–6^ M EBL improved the chlorophyll content by 24.20, 22.48 and 23.32%, net photosynthetic rate (P_*N*_) by 32.88, 32.61 and 33.61%, transpiration rate (*E*) by 16.58, 13.22 and 14.13%, *gs* by 50, 50 and 50%, intercellular carbon dioxide concentration (*C*_*i*_) by 10.52, 6.81 and 8.33%, carbonic anhydrase (CA) activity by 33.33, 25.17 and 30.06% and NR activity by 100, 81.81 and 90% in Kukrail, Tushar and Pranjal respectively as compared with their respective controls (Figs. 2A–F, 3A). The spray treatment of EBL at 10^–6^ M maximally increased the leaf nitrogen (N) content by 42.69, 50.41 and 43.19%, P content by 15.62, 17.85 and 16.12% and K content by 14.50, 12.69 and 13.61% in Kukrail, Tushar and Pranjal respectively over their respective water-treated plants (Fig. 3B–D).

### Yield and quality characteristics of peppermint are enhanced by EBL treatment

**Figure 4:**
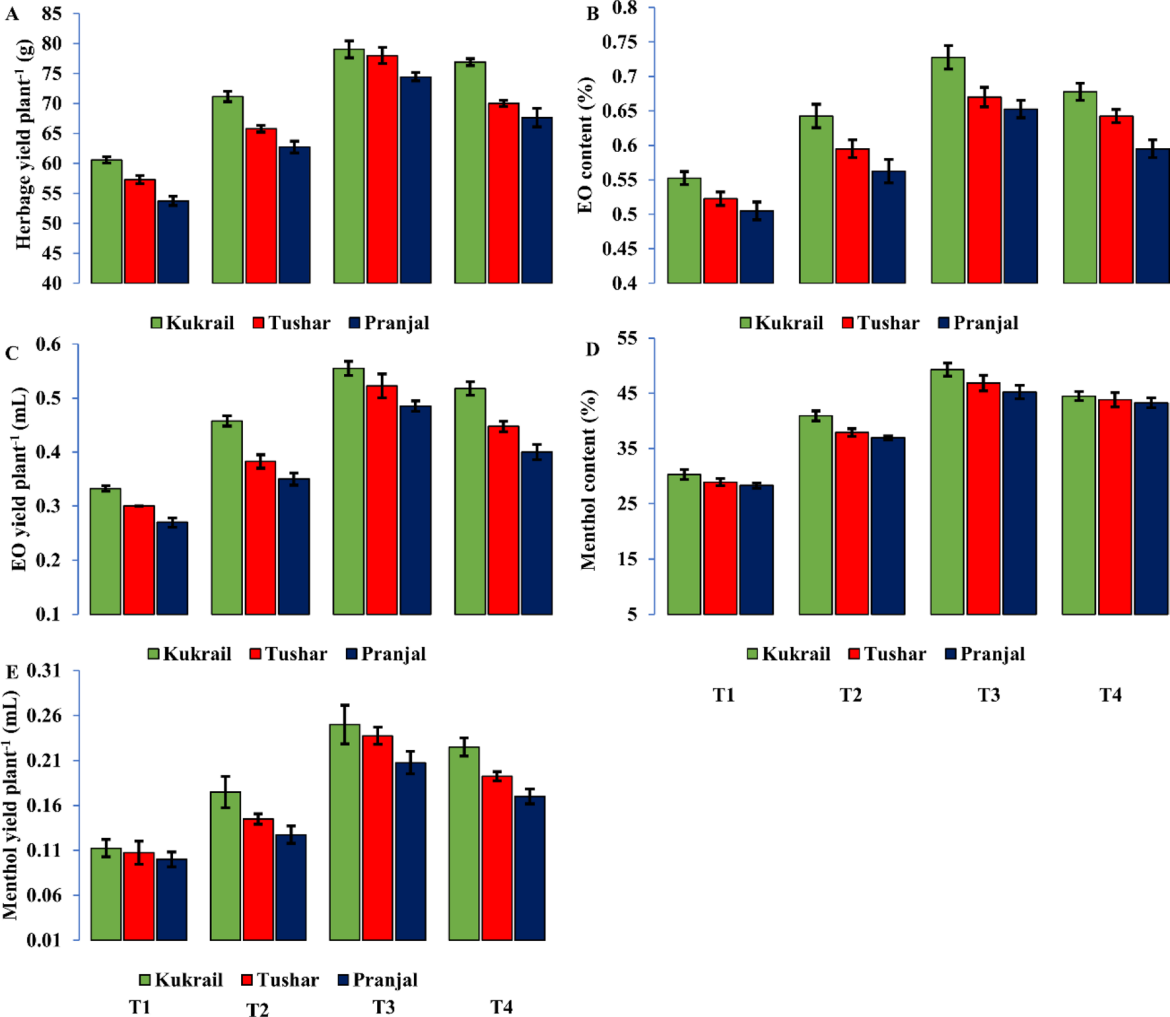
Effect of different concentrations of EBL (**A**) Herbage yield per plant (**B**) EO content (**C**) EO yield per plant (**D**) Menthol content (**E**) Menthol yield per plant of studied cultivars of Kukrail, Tushar and Pranjal. Columns represent the mean and bars represent the standard error (SE) of four replicates of each cultivar.

The exogenous application of EBL enhanced the yield attributes of all three cultivars. The three levels of EBL, particularly 10^–6^ M improved the yield attributes of three cultivars over control treatments. The cultivar Kukrail proved best and was followed by the cultivar Tushar whereas the cultivar Pranjal performed the least. The 10^–6^ M EBL enhanced the herbage yield per plant by 30.49, 28.92 and 36.17%, EO content by 32.72, 30.00 and 28.84% and EO yield per plant by 66.66, 77.77 and 73.33% in cultivar Kukrail, Tushar and Pranjal, respectively compared with their respective control plants (Fig. 4A–C).

### The volatile composition of peppermint is impacted by the foliar application of EBL

The GC–MS analysis was performed to analyse the EO content of three cultivars of peppermint. However, only some important compounds were evaluated for analysis with respect to EBL foliar application. The cultivar Kukrail proved best with an increase of 62.78% menthol content at 10^–6^ M, followed by Tushar and Pranjal with an increase of 59.99% and 62.16% respectively, as compared with their respective controls (Fig. 4D). The menthol yield per plant was enhanced by 127.27, 110 and 118.18% in cultivars Kukrail, Tushar and Pranjal, respectively compared with their respective control treatments (Fig. 4E). Apart from the enhancement in the primary compound i.e., menthol, the 10^–6^ M EBL application also resulted in improving the menthyl acetate, limonene and eucalyptol content. However, the menthone, menthofuran and pulegone were reduced with the application of EBL in all three cultivars of peppermint over their respective control treatments (Table 1).

**Table 1:** Similar compounds identified in the oil content of cultivar Kukrail receiving the treatment of T1 and T3 by GC–MS analysis.

S. No	Retention time	Name of the compound	Peak area %	
			T1	T3
1	20.17	Limonene	4.70	6.69
2	14.85	Eucalyptol	3.05	4.29
3	24.87	Menthyl acetate	6.73	6.92
4	23.09	Pulegone	2.52	1.99
5	21.03	Menthol	30.28	45.28
6	20.18	Menthone	12	5.64
7	19.52	Menthofuran	6.6	4.2
8	29.26	Caryophyllene	0.24	0.21
9	29.65	pinene	0.20	0.15

### Microscopical examination

#### Scanning electron microscopy

**Figure 5:**
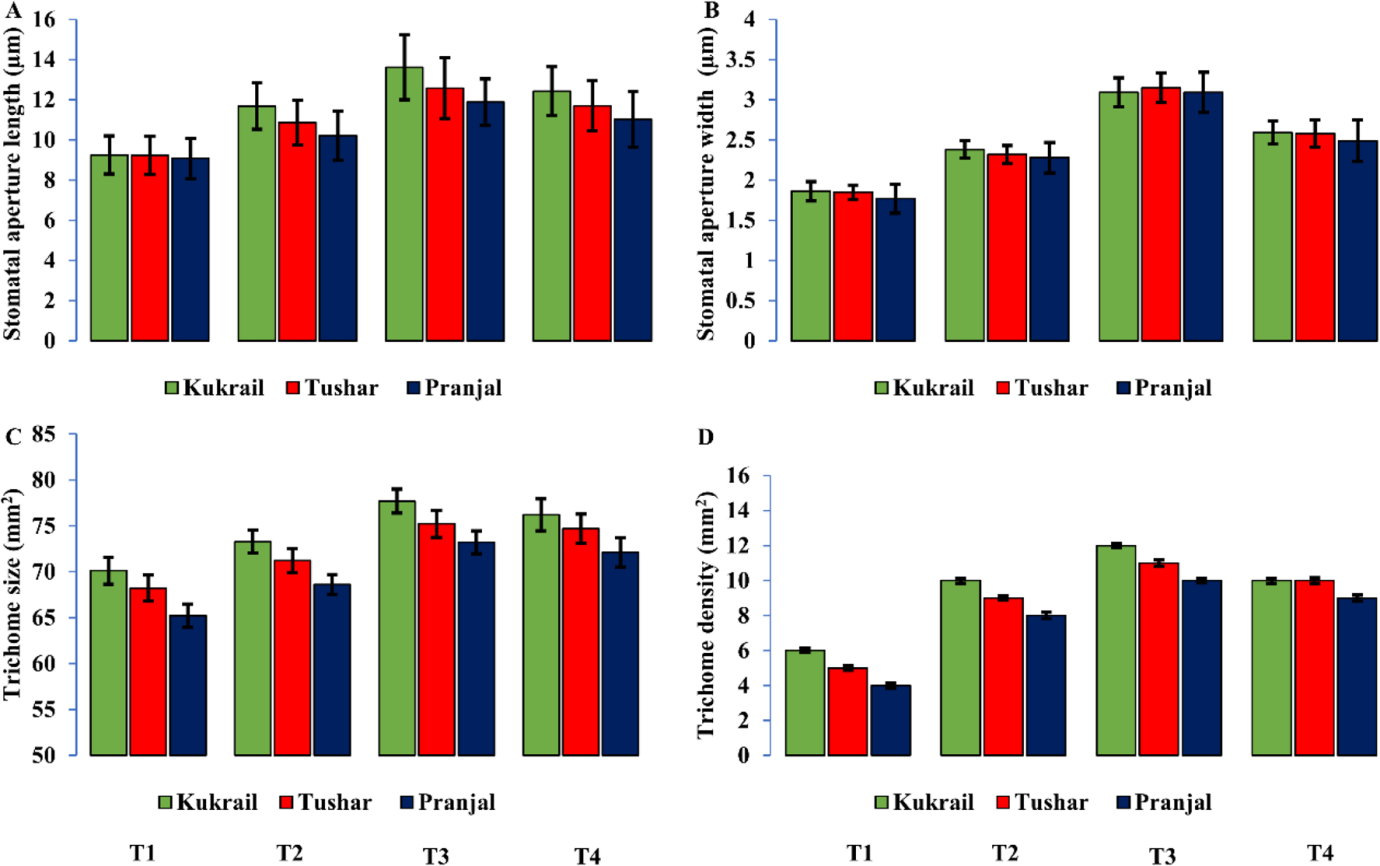
Effect of different concentrations of EBL on: (**A**) Stomatal aperture length (**B**) Stomatal aperture width (**C**) Trichome size (**D**) Trichome density of peppermint cultivars Kukrail, Pranjal and Tushar. Columns represent the mean and bars represent the standard error (SE) of four replicates of each cultivar.

**Figure 6:**
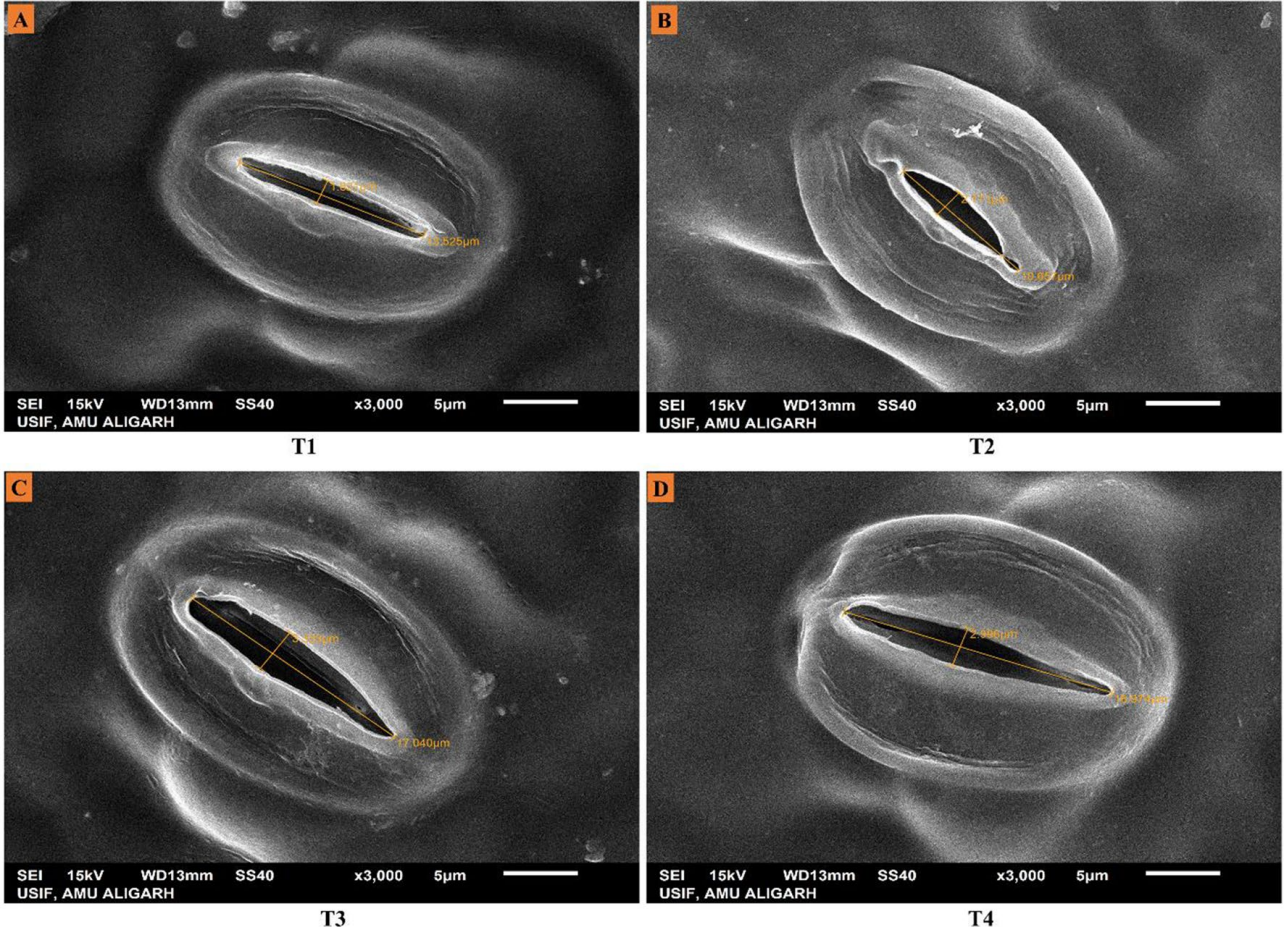
Scanning electron microscopical (SEM) images showing the stomatal behaviour of peppermint leaves. The SEM images depict an increase in the length and width of the stomatal aperture in the best performer Kukrail cultivar by EBL application. Stomatal aperture size (**A**) at (T1) (**B**) at T2 (**C**) at T3 (**D**) at T4.

**Figure 7:**
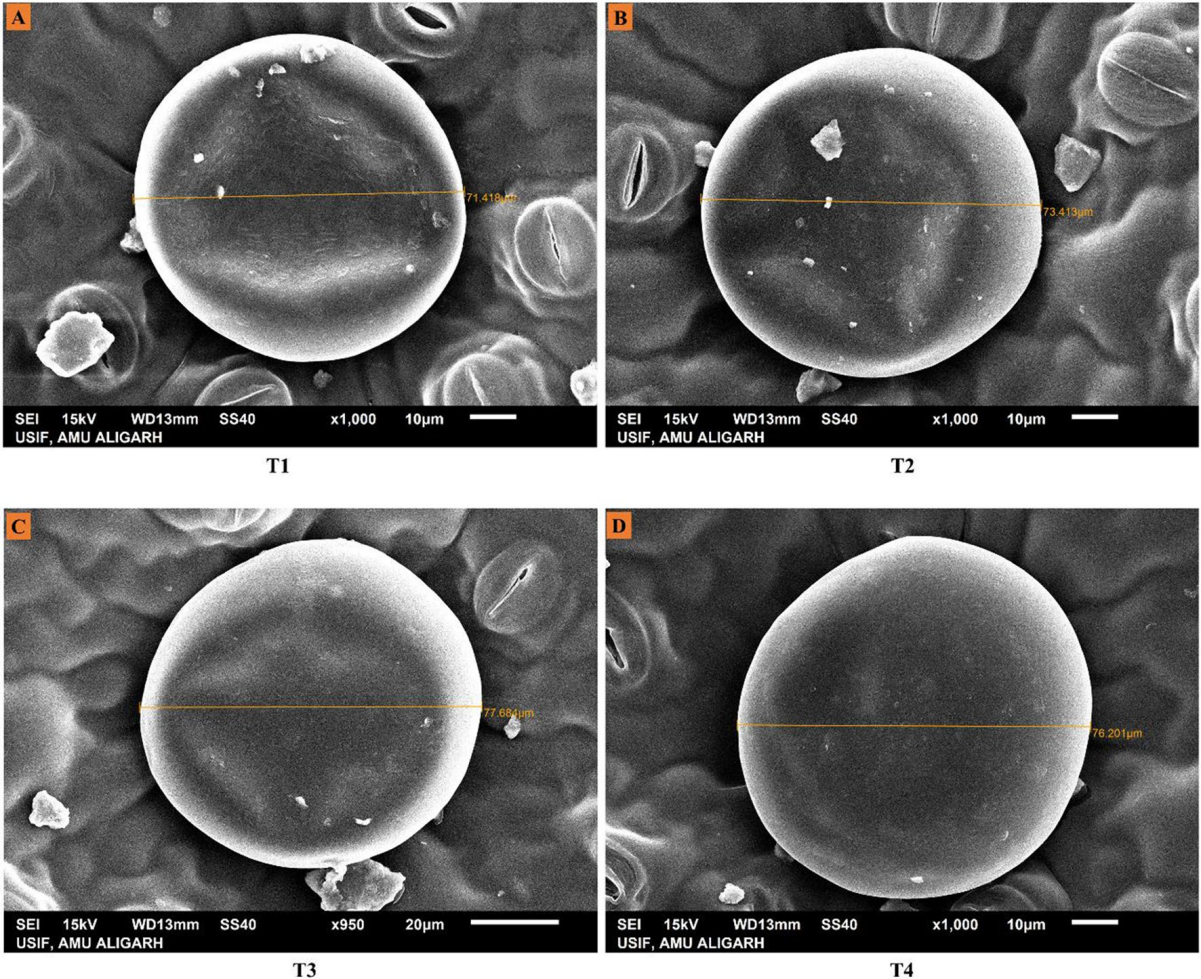
Scanning electron microscopical images (SEM) showing the trichome size of peppermint leaves. The SEM images depict an increase in trichome size in the best performer cultivar Kukrail by EBL application. Trichome size (**A**) at (T1) (**B**) at T2 (**C**) at T3 (**D**) at T4.

**Figure 8:**
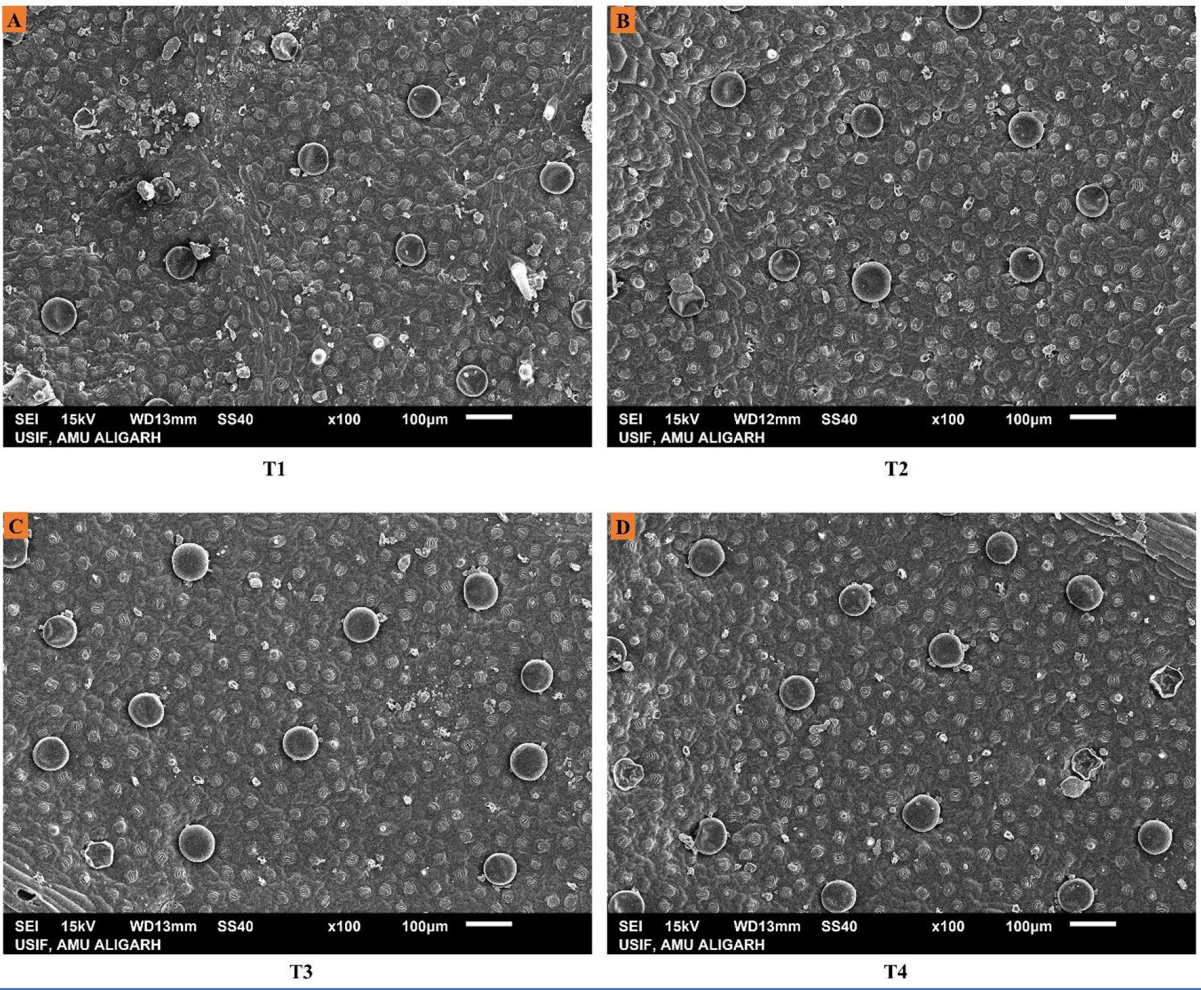
Scanning electron microscopical images showing trichome density of peppermint leaves. The SEM images depict an increase in the density of trichomes by EBL application in the best performer cultivar Kukrail. Trichome density (**A**) at (T1) (**B**) at T2 (**C**) at T3 (**D**) at T4.

The stomatal dimensions and trichome behaviour were studied under Scanning Electron Microscopy (SEM) and analysed by ImageJ software. The SEM study depicted that the stomatal aperture size (length and width) and the size and density of trichomes were found to be increased by the foliar spray of EBL. The highest values for these parameters were observed in cultivar Kukrail followed by Tushar and Pranjal. The observed mean ± SD of stomatal aperture length and width of Kukrail plants treated with 10^–6^ M was 17.00 ± 1.62 µm and 3.28 ± 0.18 µm respectively whereas, in its respective control plants the mean ± SD of stomatal length and width was 9.52 ± 2.25 µm and 1.82 ± 0.12 µm respectively (Fig. 5A,B). The observed mean ± SD of trichome size and density of Kukrail at 10^–6^ M was 77.50 ± 1.31 µm and 11 ± 0.12 mm^2^ respectively, whereas, in its respective control the mean ± SD of trichome size and density was 70.41 ± 1.45 µm and 6 ± 0.12 mm^2^ respectively (Fig. 5C,D). The representative microscopic images of the cultivar Kukrail are shown in (Figs. 6, 7, 8).

### Confocal laser scanning microscopy

**Figure 9:**
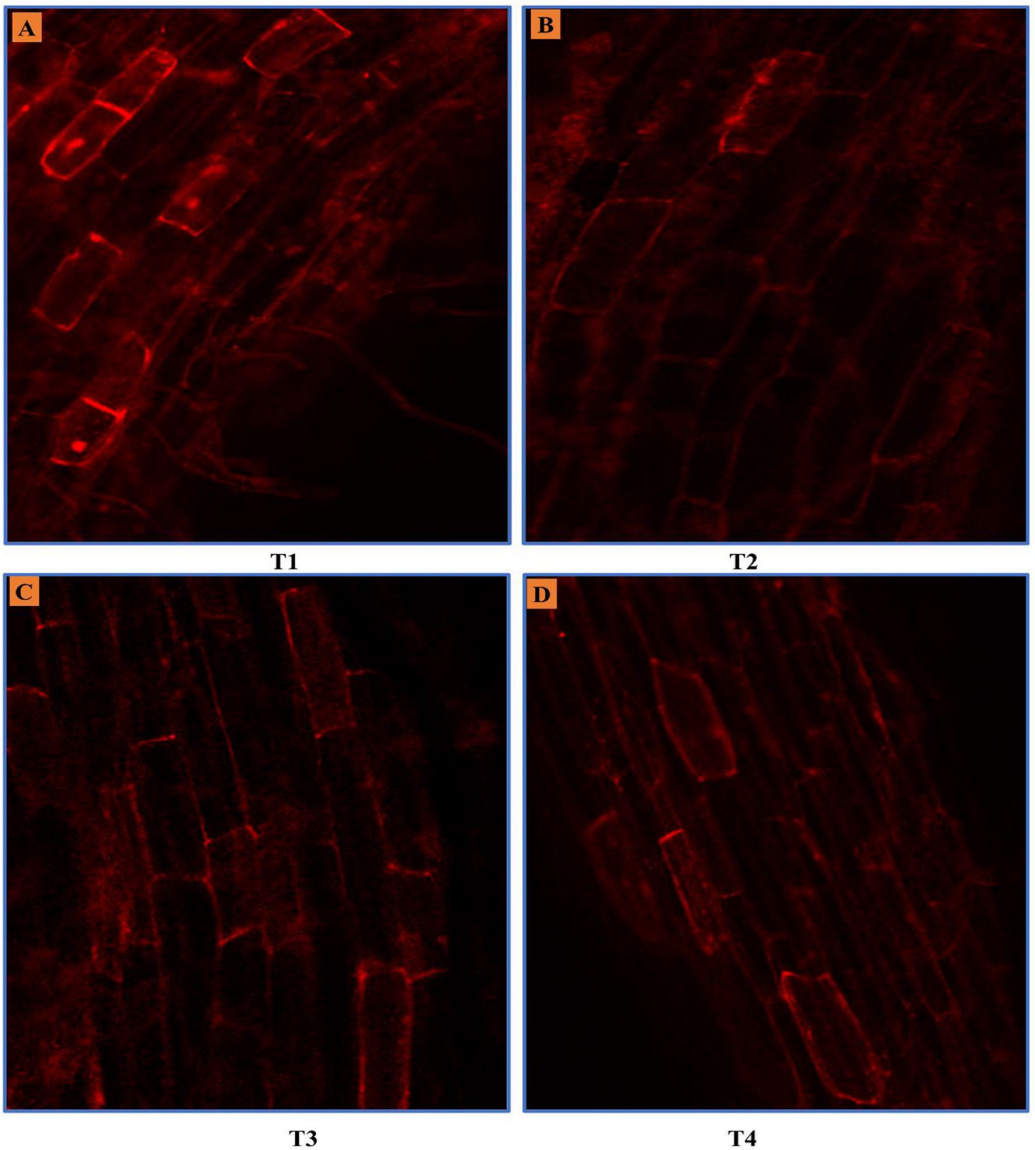
Confocal microscopical images showing the cellular viability of root cells of best performer cultivar Kukrail. Cellular viability at T1 depicts the higher fluorescence deep into the cell representing a large number of dead cells. Whereas, cellular viability at T2, T3 and T4 having the least fluorescence depict the enhanced cellular viability by EBL application, particularly at T3 treatment.

Propidium iodide is a red-fluorescent intercalating dye that is frequently used to stain cells and nucleic acids to visualise cellular viability. Propidium iodide does not gains entry into living cells due to the semipermeable cell membrane. Red fluorescent spots are produced as it enters into the dead cells through the damaged portion of the cell membrane and binds to the DNA by intercalating between the bases. In our investigation, the three cultivars of peppermint receiving the foliar treatment of EBL exhibited the least fluorescence, indicating higher root cellular viability in comparison to their respective controls. However, the cultivar Kukrail had the maximum cellular survival followed by Tushar and Pranjal. The representative confocal images of cultivar Kukrail at various treatments of EBL are given in (Fig. 9).

### Principal component analysis and heat map analysis

**Figure 10:**
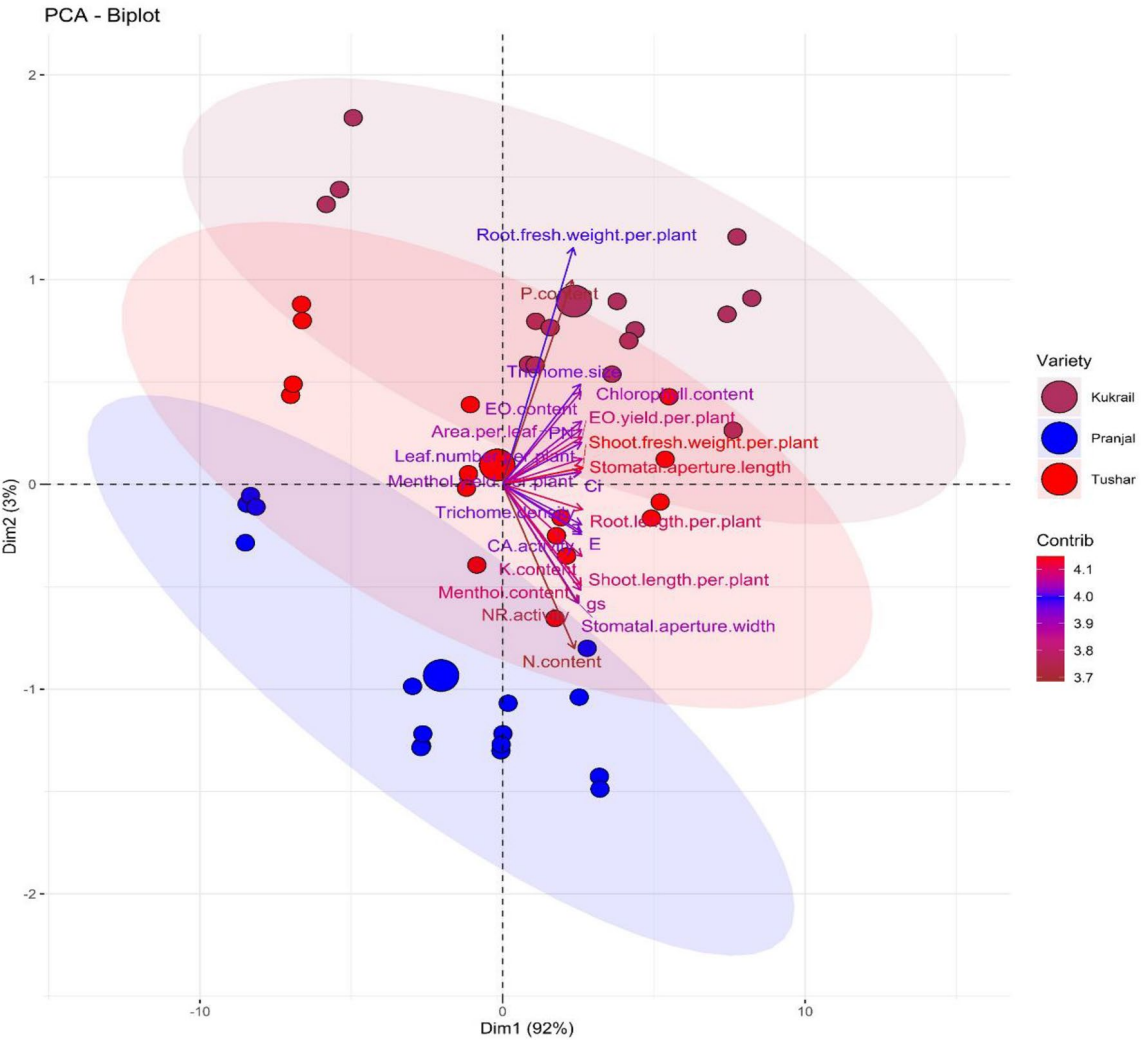
Principal component analysis (PCA) shows the EBL treatment effect on the performance of three cultivars (Kukrail, Tushar and Pranjal) of peppermint.

**Figure 11:**
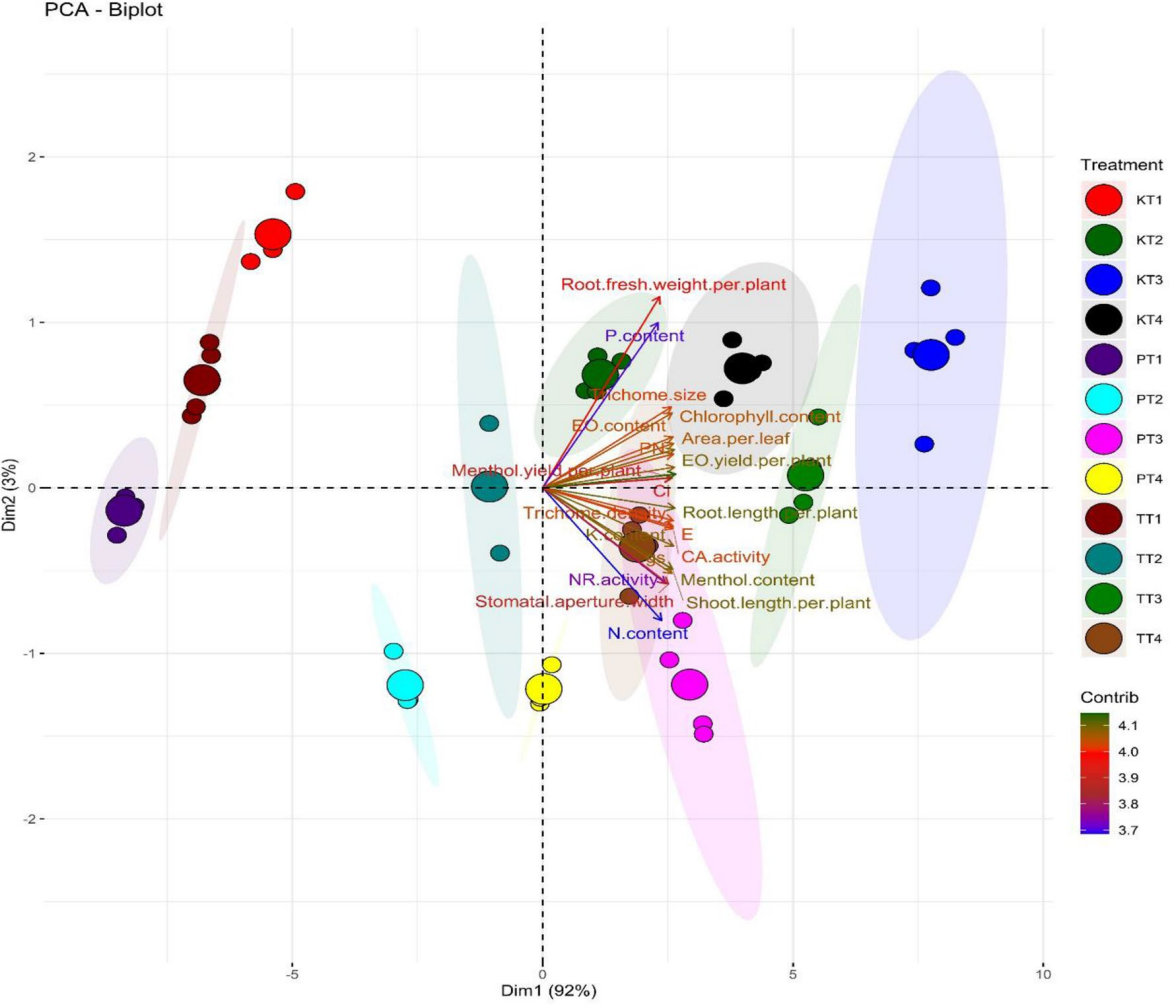
Principal component analysis (PCA) showing the interaction effect (treatment × cultivar) on the performance of three cultivars of peppermint on different levels of EBL application. KT1–KT4; cultivar Kukrail at different concentrations of EBL, PT1–PT4; cultivar Pranjal at different concentrations of EBL, TT1–TT4; cultivar Tushar at different concentrations of EBL.

Principal component analysis revealed that the data is segregated maximum by principal component 1 (Dim 1) i.e., 92% and principal component analysis 2 (Dim 2) by 3%. Principal component analysis was performed to evaluate the effect of the foliar spray of EBL on all the studied parameters. The variability in the respective parameters was evaluated with respect to varieties (Fig. 10) and treatments (Fig. 11). The Biplot formed (Fig. 10) revealed that cultivar Tushar has the maximum variability in comparison with the cultivars Kukrail and Pranjal. Although, both the cultivars Kukrail and Pranjal are segregated in the score plot. However, many variables of the cultivar Tushar overlap with the cultivars Kukrail and Pranjal. The loadings plot revealed that the leaf N, P and K content and NR activity contributed the least, whereas, shoot fresh weight per plant and stomatal aperture length contributes the most in the segregation of three cultivars. The Biplot formed with respect to individual treatments in the three cultivars (Fig. 11) revealed that all the treatments segregate in the score plot. However, the foliar spray treatment of 10^–6^ M in all three cultivars with respect to their control treatments was segregated completely.

**Figure 12:**
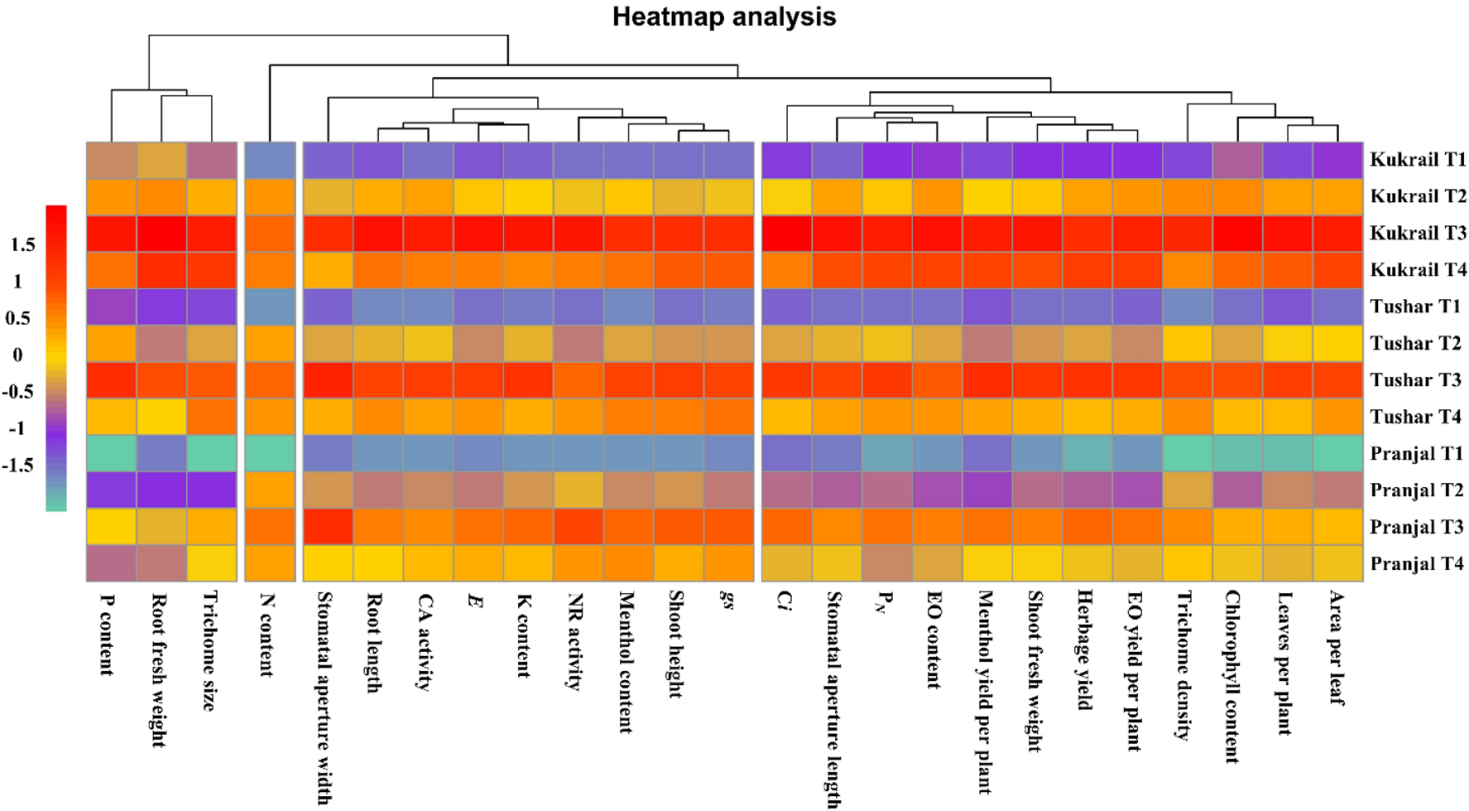
Heat map analysis showing the clustering of studied parameters of the three cultivars (Kukrail, Tushar and Pranjal) of peppermint.

The heat map analysis was performed in order to evaluate the clustering pattern of the studied parameters with respect to the foliar application of EBL in the three cultivars studied. Heat map analysis (Fig. 12) revealed that the leaf N content evaluated formed the solitary cluster, whereas, trichome size, root fresh weight per plant and the leaf P content formed the separate cluster. The stomatal aperture width, root length, CA activity, NR activity, *E*, leaf K content, menthol content, shoot length and *gs* were found to form another cluster. The remaining 12 parameters were found to cluster into a common group based on Euclidian distance among them with respect to the foliar application of EBL.

### Correlation analysis between traits

**Figure 13:**
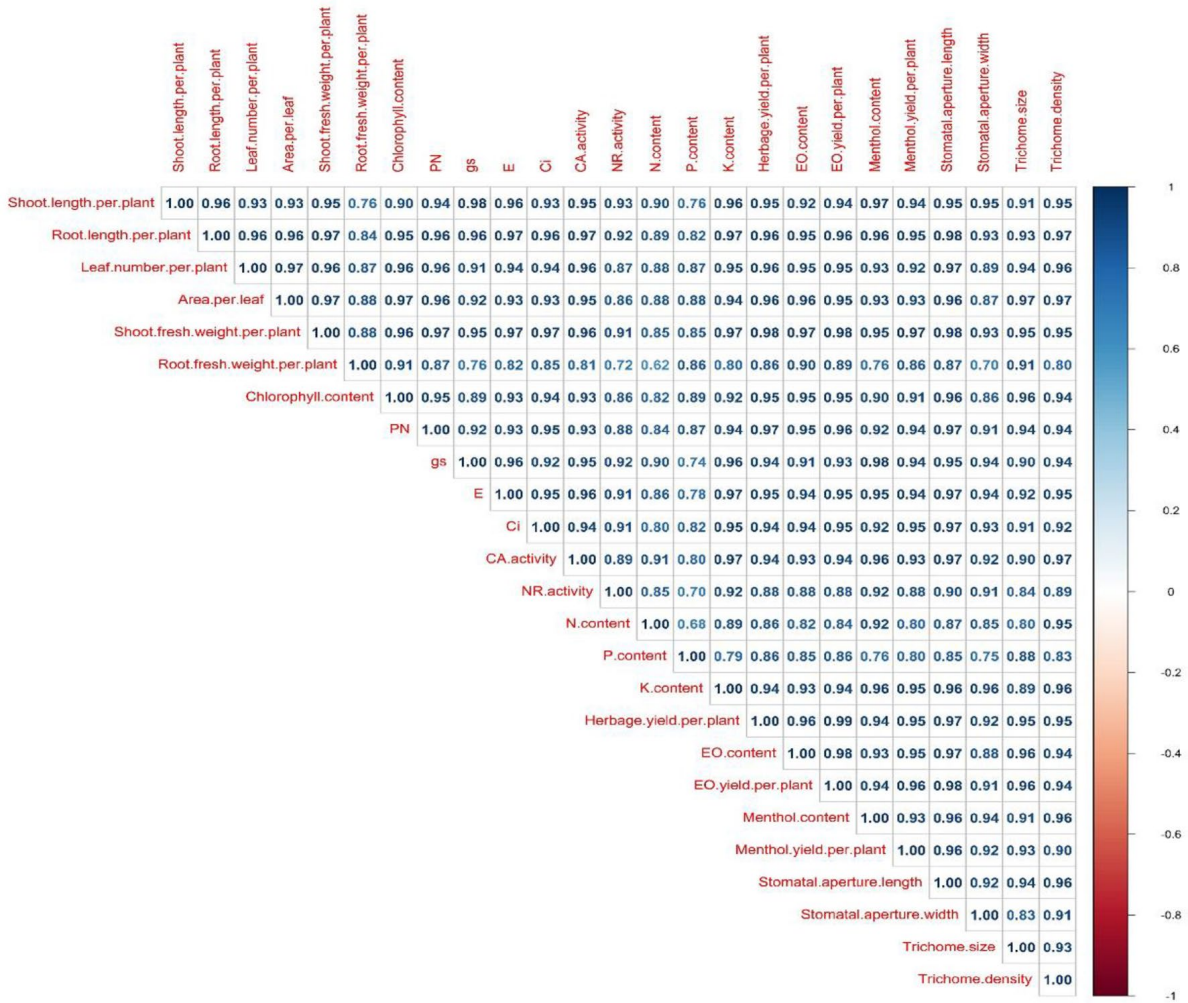
Pearson correlation analysis among the studied parameters of peppermint cultivars.

Pearson correlation analysis was performed at a 95% significance level. The correlation was performed among all the studied parameters in all three cultivars of peppermint. The correlation between menthol content and shoot fresh weight per plant showed a correlation with r0.05 = 0.95, trichome size and EO content with r0.05 = 0.96, trichome size and menthol content with r0.05 = 0.91, trichome density and EO content with r0.05 = 0.94 and trichome density and menthol content with r0.05 = 0.96. Therefore, from this study, it can be stated that these parameters are closely associated with each other (Fig. 13).

## Discussion

In the present study, the response of three cultivars of peppermint by the foliar application of EBL was observed. The survey of data revealed that the foliar application of EBL improved all the growth parameters studied. The increase in shoot and root length per plant, leaf number per plant, area per leaf and leaf area per plant may be attributed to the roles of EBLs in plants. The growth-promoting effect may be due to the role of brassinosteroids in upregulating the gene expression of xyloglucan endotransglycosylases which are involved in cell wall loosening for cell elongation and CycD3 gene (D-type plant cyclin) which play a promotive role in cell division and cell slackening^16, 17^. Besides, EBLs have a prominent role in cell and tissue differentiation and organogenesis that would have culminated in an increased shoot and root fresh weight, hence higher values for the shoot and root fresh weight. Our results are supported by the findings on *Coleus forskohlii* L.^18^, *Mentha arvensis* L.^19^ and *Lavandula intermedia* L*.*^8^.

The exogenous application of EBL improved chlorophyll content, gas exchange parameters (P_*N*_, *gs*, *Ci* and *E*), CA activity, NR activity and leaf N, P and K contents over water spray treatments (Figs. 2, 3). The enhancement in chlorophyll content may be due to the involvement of EBL in the expression of genes responsible for the synthesis and/or activation of the enzymes responsible for chlorophyll biosynthesis and other associated light-harvesting complex proteins^20^ and minimizing the rate of chlorophyll degradation^21^. The increase in gas exchange parameters may be assigned to the role of EBL in upregulating the gene expression of the RuBisCo large subunit gene (rbcL), RuBisCo small subunit gene (rbcS), RuBisCo activase gene and other photosynthetic genes. The upregulated expression of these genes enhances the RuBisCo carboxylation rate (*V*c max) which in turn promotes the catalysis of Ribulose bisphosphate for carboxylation during the Calvin cycle^22^. Epibrassinolides promote stomatal conductance thereby facilitating the diffusion of carbon dioxide into the stomatal cavity. Moreover, the EBL is known to upregulate the key enzymes of the Calvin cycle such as triose-phosphate isomerase, glycerate phosphate-3-kinase, fructose 1,6-bisphosphate, sedoheptulose-1,7-bisphosphate and ribulose-5-phosphate kinase^23, 24^. Epibrassinilode plays a significant role in increasing the carbon dioxide assimilation rate and quantum yield of photosystem II (PS II)^14^. Consequently, the enhanced carbon dioxide assimilation rate and RuBisCo activity would have enhanced the Calvin cycle efficiency. Thus, EBL-mediated cumulative direct or indirect upregulation in the expression of photosynthetic-related genes and carbon dioxide assimilation rate would have led to enhanced photosynthetic-related parameters. Our results are in accordance with the reports on *Mentha arvensis* L.^19^, *Ferula assafoetida* L.^25^, *Vigna unguiculata* L.^26^ and *Brassica juncea* L.^27^.

In this study, the foliar application of EBL promoted the CA and NR activity of peppermint (Figs. 2F, 3A). Carbonic anhydrase catalyzes the reversible interconversion of bicarbonate (HCO_3_^-^) and carbon dioxide and is found in close association with RuBisCo by increasing the concentration of carbon dioxide at its active site. The EBL play a significant role in the activation of CA activity which might be due to the upregulation of the CA gene expression^28^. The EBL-promoted CA activity has also been reported on *Mentha arvensis* L.^19^ and *Catharanthus roseus* L.^29^. The increase in the NR activity of peppermint may be due to the enhanced transcription or translation of NR-related genes. The observation is in agreement with the reports on *Solanum lycopersicum* L.^30^ and *Brassica juncea* L.^27^.

Epibrassinolides are involved in the root elongation processes in plants. Moreover, the EBL signalling cascade activation promotes the NH_4_^+^ uptake by modulating the expression pattern of the *ammonium transporter 1* (*AMT1*) gene in *Arabidopsis thaliana* ^31^. Besides the EBL application upregulates the gene expression of *nitrate transporter genes* (*NRT/NPF*) such *as ZmNRT2.1, ZmNRT2.2, ZmNPF6.4* and *ZmNPF6.6* which leads to the N uptake in *Zea mays* L.^32^. That might have led to increasing the content of N in treated plant leaves. Moreover, it has been reported that the EBL influences long-distance signalling by regulating the auxin transport^33^. Probably this might have interfered with the activities of mineral element parameters and hence higher values for leaf N, P and K in EBL-treated plants (Fig. 3B–D). Our findings also corroborate the results of^29^ on *Catharanthus roseus* L. and^34^ on *Trigonella foenum-graecum* L.

In our study, it was found that the foliar spray of EBL increased the stomatal dimensions of peppermint cultivars (Fig. 5A,B). The observed change mediated by the EBL might be due to the fact that EBL has a prominent role in the upregulation of the K^+^ ion channel which enhances the K^+^ uptake in guard cells^35^. Therefore, EBL-regulated K^+^ ion channels may be responsible for increasing the stomatal dimensions of EBL-treated plants over control.

In our study, it was observed that the foliar application of EBL increased the glandular-secreting trichome (GST) size and density of peppermint (Fig. 5C,D). The observed changes might be due to the role of EBL to control trichome development directly or indirectly by interfering with the trichome developmental regulators like TRANSPARENT TESTA GLABRA1 (*TTG1*), GLABRA1 (*GL1*) and ENHANCER OF GLABRA3 (*GL3/EGL3*). These three genes form an activator trimeric complex that activates the expression of downstream target genes like *GL2* and *TTG2* which in turn regulates the trichome initiation^36^. The involvement of brassinosteroids in the development of GSTs has also been reported earlier in *Arabidopsis thaliana* L.^37^.

In this study, the foliar application of EBL increased the root cell viability of peppermint (Fig. 9). The enhanced cell viability of roots may be due to the role of EBL in promoting cell expansion and maintaining the constant cell number in the root meristem^38^. Exogenous application of EBL is known to upregulate the gene expression of *ZmWOX5*, *ZmBBM1*, and *ZmBBM2*; necessary for the formation of stem cell microenvironment in the root meristem^39^, *ZmPIN2*, *ZmPIN3a* and *ZmPIN3b*; maintains the activity of root apical meristem^40^, *ZmARF7* and *ZmARF19*; factors promote the initiation of lateral root development^41^. The role of exogenously applied EBL to stimulate root growth has been reported in *Arabidopsis thaliana* L.^42^.

In this study, EO content and yield were significantly increased by EBL applications (Fig. 4). The highest EO content and yield were obtained at 10^–6^ M EBL in all the cultivars studied. The increasing effects of EBL on EO content are attributed to stimulative influences on the intrinsic genetic makeup associated with EO production^19^. The increase in the EO may also be due to the role of EBL in increasing the activity of linalool synthase and linalool acetyltransferase involved in the biosynthetic pathway of EOs^8^. Besides, the change in the peppermint oil content and quality may be due to the effect of EBL on key enzymes such as geranyl diphosphate synthase, limonene synthase, limonene-3-hydroxylase, trans-isopiperitenol dehydrogenase, isopiperitenone reductase, cis-isopulegone isomerase, pulegone reductase and menthone reductase involved in the EO biosynthetic pathway^4^. Moreover, according to^43^ photosynthesis and EO production have a positive relationship. It is well known that EBL promotes chlorophyll and photosynthesis in plants^4, 19^. Based on the relationship between photosynthesis and oil production, it is possible to conclude that the promoting effects of EBL on EO production may be related to its increasing effects on photosynthesis. Moreover, the increase in size and density of GSTs of EBL-treated peppermint would also have enriched the EO production due to their role in biosynthesis, secretion and accumulation of EO. Our results are in accordance with the findings in *Pelargonium graveolens* L.^18^, *Mentha piperita* L.^4^, *Lavandula intermedia* L.^8^ and *Ferula assafoetida* L.^25^.

## Conclusion

The current research suggests that the exogenous application of EBL improved the overall performance in terms of growth, productivity, quality and quantity of EO content of the three studied peppermint cultivars. Among the treatments, 10^–6^ M EBL application promoted the overall performance of the studied cultivars whereas, the positive effect was slightly weakened at 10^–7^ M. The cultivar Kukrail proved best followed by Tushar and Pranjal. The better performance of the Kukrail cultivar may be due to the better genetic makeup which might have been further enhanced by the application of EBL treatment. The investigation indicates that 10^–6^ M EBL application is effective in increasing the crop productivity, the nutritional content of the leaf as well as the biochemical and qualitative features of the peppermint crop that increases its economic importance in the world market. Our findings were supported by microscopical studies and GC–MS analysis. Future strategies for EBL application in crop plants should focus on understanding its molecular mechanisms in specialized metabolite biosynthesis and deciphering the genes and pathways affected to enhance desired compound production. Sustainable farming practices incorporating EBL can maximize crop performance, increase yields, and improve the quality of valuable plant products, benefiting farmers economically and ensuring long-term agricultural productivity and environmental integrity, thus enhancing competitiveness in the global market.

## Materials and methods

### Experimental plant material

The healthy and fresh suckers of peppermint cultivars were used in experimental work. The plant material was procured from the Central Institute of Medicinal and Aromatic Plants, Lucknow, India (CIMAP). The experimental work was started after seeking permission from the Department of Botany Aligarh Muslim University (AMU), Aligarh and also with the consent of the CIMAP Lucknow (India). After collecting the plant material, they were then transplanted into the earthen pots for further study. The whole experiment was performed in accordance with the relevant rules and regulations of AMU. A preliminary experiment was carried out to assess various growth and physiobiochemical parameters of peppermint cultivars. Based on these parameters, the best three superior cultivars, Kukrail, Pranjal and Tushar were selected for further experimental purposes.

### Experimental design and treatment pattern

A pot experiment was performed in a net house of the Department of Botany, Aligarh Muslim University, Aligarh India during the summer season. The earthen pots (25 × 25 cm) were filled with 5 kg of a mixture of clay soil and organic manure in a ratio of 4:1. The healthy, fresh and uniform suckers were carefully transplanted into the earthen pots at the rate of 3 suckers per pot. At the time of transplanting, a recommended basal dose of N, P and K were applied to the soil at 36 mg N, 17.9 mg P_2_O_5_ and 17.9 mg K_2_O kg^-1^ soil, i.e., 80 kg N + 40 kg P_2_O_5_ + 40 kg K_2_O ha^-1^^44^. The half dose of N and full doses of P and K were applied to the soil at transplanting of the suckers and the remaining half dose of N was top-dressed at 30 days after transplanting (DAT). Nitrogen, P and K were applied in the form of urea, single super phosphate and muriate of potash, respectively. The experiment was conducted according to a factorial randomised design with four replicates. A total of 60 earthen pots were used and these pots were grouped into 3 sets of 20 pots each, with one set of pots being assigned to respective cultivars. Watering and weeding were undertaken as and when required. The foliage of plants was sprayed with EBL at 0 (control; T1), 10^–5^ M (T2), 10^–6^ M (T3) and 10^–7^ M (T4) twice by using a hand sprayer with the first spray being given at 60 DAT (spray time 9:20 am, average temperature 26 °C and relative humidity 30%) and the second spray at 80 DAT (spray time 9:20 am, mean day temperature 29 °C and relative humidity 24%). Growth, physio-biochemical and microscopical parameters were studied at 100 DAT and yield and quality attributes at 120 DAT.

### Determination of growth attributes

To determine the phenotypical characteristics of peppermint cultivars, one grown sucker from each earthen pot was carefully uprooted and washed carefully with tap water to clean the root surface. The length of the root and shoot was measured with the help of a metric scale and expressed in cm. The area per leaf of the upper third fully expanded leaf was measured by using graph paper and expressed in cm^2^. The leaf number per plant was noted manually. Leaf area per plant was computed on the basis of leaf number per plant and area per leaf. The fresh weight of the shoot and root was measured with the aid of digital balance and expressed in g.

### Physiobiochemical parameters

#### Chlorophyll content

The chlorophyll content of intact leaves was measured at 11:00 a.m. in natural environmental conditions on a full sunny day using a chlorophyll metre SPAD-502 (KMS Inc. Japan).

### Gas exchange parameters

Parameters related to gaseous exchange, viz. *C*_*i*_, P_*N*_, *g*_*s*_ and *E* were determined on the upper sixth completely grown leaves of the main axis of all plants by using an Infrared Gas Analyzer (IRGA) Portable Photosynthesis System (LI-COR-6400, Lincoln, Nebraska, USA). The photosynthetic measurements were noted within a minute after closing the leaf chamber. The measurements were recorded at 11:30 am on a full sunny day at a mean temperature of 30 °C.

### Carbonic anhydrase activity

The CA activity was estimated using Dwivedi and Randhawa’s procedure^45^. Young plant leaves were chopped into small pieces and placed into Petri plates containing an aqueous 10 mL cysteine hydrochloride solution (0.2 M). The leaves were incubated for about 20 min at 4 °C followed by the removal of the solution from the leaf surface with blotting paper. The pieces were then put into a reaction vessel followed by the addition of 4 mL phosphate buffer (pH 6.8), 4 mL of 0.2 M sodium bicarbonate (NaHCO_3_) and 0.2 mL of 0.002% bromothymol blue indicator. The reaction vessel was then stirred and incubated at 4 °C for 20 min. After that, the reaction mixture was titrated against 0.05 N hydrochloric acid (HCl) using methyl red as an indicator. Finally, the enzyme activity was expressed in terms of µmol CO_2_ kg^-1^ FWs^-1^.

### Nitrate reductase activity

Nitrate reductase activity was studied by using the method of^46^ and its activity was expressed in µmol NO_2_ g^-1^ FM s^-1^.

### Leaf element contents (N, P and K)

Leaf samples were wrapped in paper and dried for 48 h in an oven maintained at 80 °C. Thereafter, these dried leaves were ground into a fine powder using a mortar pestle. The 100 mg leaf powder of each sample was transferred into a digestion tube to which 2 mL of analytical reagent grade concentrated sulphuric acid was added. The digestion tube was then heated for about 2 h on a Kjeldahl assembly at 80 °C, followed by cooling the reaction mixture at room temperature for about 15 min. Thereafter, 0.5 mL of 30% H_2_O_2_ was added dropwise to the cooled mixture followed by mild heating (at 50 °C) till the colour of the solution turns light yellow. Again, after cooling, 4–5 drops of 30% H_2_O_2_ were added followed by gentle heating. The addition of 30% H_2_O_2_ was repeated until the reaction mixture turns colourless. This reaction mixture containing the peroxide-digested leaf material was used to evaluate leaf N, P and K contents. The leaf N and P contents were estimated following the methods of^47, 48^ respectively. Leaf K content was determined using a flame-photometer (Model: C150, AIMIL, India) by adopting the method of^49^. The N, P and K content of the leaf was expressed in terms of percentage (%) on a dry weight basis.

### Microscopical examination

The microscopical examination was performed in the samples of three cultivars receiving the foliar treatment of EBL.

### Confocal microscopy

Thin transverse sections of root samples were soaked in propidium iodide dye for 20 min. The root samples were then taken out and washed with double distilled water. Thereafter, the stained roots were mounted on a glass slide with forceps and examined under a confocal laser scanning microscope (Zeiss, LSM 780, Germany).

### Scanning electron microscopy

The stomatal and trichome behaviour of the freeze-dried leaf samples was investigated using SEM (JEOL, JSM 6510 LV, Japan). Leaf samples were dissected and fixed in a solution of 2% formaldehyde (1 mL) and 2.5% glutaraldehyde (98 mL) and 100 mM sodium cacodylate (1 mL) in phosphate buffer (100 mM, pH 7.3) for 120 min. The leaf samples were then dehydrated using an ethanol series (30%, 50%, 70%, and 100%). The dehydrated samples were then cut into strips and besmeared with gold–palladium and the trichome exterior appearance was studied at the magnification of 100× and 1000× whereas the stomatal dimensions were observed at 3000× at 15 kV.

### Yield and quality characteristics

Yield and quality parameters such as herbage yield per plant, EO content, EO yield per plant, menthol content and menthol yield per plant were evaluated at harvest (120 DAT).

### Essential oil extraction and compositional analysis

The remaining two grown suckers of a plant were used for determining herbage yield per plant. Essential oil of peppermint was extracted using Clevenger’s apparatus (Borosil, India). Fresh leaf samples (50 g) were taken and chopped into little pieces. Leaf samples and 500 mL tap water were placed in a flask connected to the condenser of the Clevenger apparatus. The hydrodistillation was undertaken for three hours. Thereafter, the EO content (%) and EO yield per plant were calculated. The extracted oil was dried with anhydrous sodium sulphate and stored at 4 °C for its gas chromatography and mass spectrometry (GC–MS).

### GC–MS analysis of essential oil

The EO was analysed using a GC-MS-TQ8050 NX (Japan) at the Central Instrumentation Laboratory of the Central University of Punjab, Bathinda, India. For GC–MS, the split injector temperature was set at 280 °C with a split ratio of 5.0. The oven temperature was programmed at 0–40 °C withheld for 3 min, increased to 220 °C at the rate of 4 °C min^-1^ and hold for 5 min. The oven temperature was further increased to 250 °C at the rate of 15 °C min^-1^ and held for 5 min. the ion source temperature was set at 230 °C and the interface temperature at 250 °C using Helium as carrier gas at a constant flow of 1 mL min^-1^. The mass scan range was set to 40–800 amu. The column flow was set at 1.00 mL min^−1^, while the column oven temperature was set at 40 °C. The sample loading/injection volume was set to 1 µL. The phytochemicals present were evaluated based on the retention time and m/z ratio using the NIST17R library and NIST17M2 library. The EO constituents were determined by computing their peak area in the chromatogram of the treatment plants over the control ones of the three cultivars.

### Statistical analysis

The data were examined statistically by using SPSS 25.0 statistical software (SPSS Inc., Chicago, IL, USA). The Pearson correlation, principal component analysis, heat map analysis and boxplot were performed using “R” statistical software and the SEM images were analysed by ImageJ software.

## Data Availability

All data generated or analysed during this study are included in this published article and are available from the corresponding author upon reasonable request.

## Abbreviations

EO: Essential oil
CA: Carbonic anhydrase
NR: Nitrate reductase
N: Nitrogen
P: Phosphorus
K: Potassium
